# Whole-Transcriptome Analysis Reveals Long Noncoding RNAs Involved in Female Floral Development of Hickory (*Carya cathayensis* Sarg.)

**DOI:** 10.3389/fgene.2022.910488

**Published:** 2022-05-11

**Authors:** Caiyun Li, Hongmiao Jin, Wei Zhang, Tao Qin, Xin Zhang, Zhenyang Pu, Zhengfu Yang, Kean-Jin Lim, Zhengjia Wang

**Affiliations:** State Key Laboratory of Subtropical Silviculture, College of Forestry and Biotechnology, Zhejiang A&F University, Hangzhou, China

**Keywords:** hickory, long noncoding RNA (lncRNA), flowering, hormones, ambient temperature, ceRNA network

## Abstract

Hickory, an endemic woody oil and fruit tree species in China, is of great economic value. However, hickory has a long juvenile period and an inconsistent flowering of males and females, thus influencing the bearing rates and further limiting fruits yield. Currently, it is reported that long noncoding RNAs (lncRNAs) play critical regulatory roles in biological processes. However, the role of lncRNAs in the development of hickory female flowers remains unclear. In this study, a total of 6,862 putative lncRNAs were identified from the female flower transcriptomes in three different growth stages of hickory. We proposed that lncRNAs might play an important role in phytohormone signaling processes for flower formation, especially in the abscisic acid and jasmonic acid pathways, according to the results of our Kyoto Encyclopedia of Genes and Genomes (KEGG) enrichment. Moreover, we predicted the interactions among four microRNAs (miRNAs), three lncRNAs, and four genes. We proposed that facing the changing environment, LNC_002115 competes with *PHOSPHATE2* (*PHO2)* for the binding sites on cca-miR399f, and protects *PHO2* from suppression. In addition, cis-acting LNC_002115 regulates the expression of the *SHORT VEGETATIVE PHASE (SVP)* by influencing *ABRE-binding factor* (*ABF*). In brief, LNC_002115 regulates hickory female floral development by impacting both *PHO2* and *SVP*. This study was the first to identify lncRNAs involved in hickory female floral development, and provided new insight to elucidate how lncRNAs and their targets play a role in female floral development in hickory, thus unfolding the opportunities for functional characterization of blossom-related lncRNAs in further studies.

## Introduction

With the advancement of DNA sequencing technology and transcriptome analysis, transcriptome studies in fungi, plants, and animals have revealed that up to 90% of the generated genomes are transcribed into RNAs (Kung et al., 2013; Ariel et al., 2015). The majority of them are found with little or no protein-coding potential, thus are called non-coding RNAs (ncRNAs) (Chekanova et al., 2007; Kapranov et al., 2007; Ariel et al., 2015). These ncRNAs are an integral and functional component of the genome. Among ncRNAs, there is long non-coding RNAs (lncRNAs), which contain more than 200 nucleotides but lack protein-coding capacity (Severing et al., 2018). LncRNAs are produced by exon, intron, intragene, intergene, promoter regions, 3′- and 5′- UTR, or coding regions in the sense and antisense directions (Nie et al., 2012). Based on their locations in the chromosomes relative to protein-coding genes, lncRNAs can be classified as antisense lncRNAs, intronic lncRNAs, divergent lncRNAs, intergenic lncRNAs, promoter upstream lncRNAs, promoter associated lncRNAs, and transcription start site-associated lncRNAs (Iyer et al., 2015; Mattick and Rinn, 2015). LncRNAs were long considered purely transcriptional “noise”; however, emerging evidence has shown that lncRNAs play important roles in key biological processes, such as genomic imprinting, gene transcription (Pruneski et al., 2011), post-transcriptional modification (Wilusz, 2016), translation (Hadjiargyrou and Delihas, 2013), X-chromosome, and the regulation of DNA methylation (Ard et al., 2014; Au et al., 2017).

In plants, although a large number of lncRNAs have been predicted in different biological contexts in quite a few plant species (Ding et al., 2012; Li et al., 2014; Shuai et al., 2014; Zhang et al., 2014; Hao et al., 2015), only a handful of plant lncRNAs have been experimentally characterized. Several lines of evidence have shown that these lncRNAs play critical roles in regulating the expression of genes involved in biotic or abiotic stress responses (Franco-Zorrilla et al., 2007; Di et al., 2014; Zhu et al., 2014; Seo et al., 2017; Song et al., 2021), vegetative growth (Ariel et al., 2014), fruit development (Bai et al., 2019), reproductive processes (Ding et al., 2012; Zhang et al., 2014; Li et al., 2019), and flowering time (Swiezewski et al., 2009; Heo and Sung, 2011; Kim and Sung, 2017) in plant life cycle. In *Arabidopsis thaliana*, about 6,480 lncRNAs were identified using genome-wide screening of 200 transcriptome data with tissue-specific expression profiles (Liu et al., 2012). In *Populus L*., computational analysis predicted potential target genes for Gibberellin (GA)-responsive lncRNAs, which take part in various biological processes that influence growth and wood properties (Tian et al., 2016). It is worth noting that lncRNAs play a key role in the regulation of *Flowering Locus C (FLC)* expression and flowering time regulation (Swiezewski et al., 2009; Hornyik et al., 2010; Liu et al., 2010; Sun et al., 2013; Marquardt et al., 2014; Wang et al., 2014). The lncRNAs COOLAIR and COLDAIR interacted with the polycomb-responsive complex 2 (PRC2) during vernalization in *A. thaliana*, further modulating vernalization-mediated epigenetic silencing of the *FLC* (Heo and Sung, 2011).

Recently, the competing endogenous RNA (ceRNA) theory has been widely studied (Salmena et al., 2011; Ala et al., 2013). The theory demonstrated that lncRNAs serve as endogenous microRNA (miRNA) sponges in the ceRNAs group (Salmena et al., 2011). As lncRNAs share common binding sites with miRNAs, they pair with miRNAs to competitively inhibit miRNA’s functioning on mRNAs (Wu et al., 2013; Paraskevopoulou and Hatzigeorgiou, 2016). In brief, lncRNAs can compete with miRNAs to regulate mRNAs’ expression; this phenomenon can be noticed in both plants and animals. For example, in *Populus L.*, potential cis-regulated and trans-regulated target genes for some lncRNAs have been experimentally demonstrated to participate in biological processes, such as tree growth and wood property formation (Liu et al., 2010). In *Solanum lycopersicum*, lncRNAs regulate the expression of tomato floral-related mRNAs by serving as ceRNAs, regulating specific bio-metabolic pathways in tomato (Yang et al., 2019). In *Zea mays L.*, seven new lncRNAs were identified, which are functional ceRNAs that affect the developmental and metabolic processes of maize seeds (Zhu et al., 2017). In *Oryza sativa L*., lncRNA osa-eTM160 attenuated the repression of osa-miR160 on *osa-ARF18* mRNA during early anther development by targeting mimicry, thus contributing to the regulation of seed size and fruit set (Wang et al., 2017). These studies have clarified the partial roles of lncRNAs. However, the role of lncRNAs in the regulation of flowering in woody plants is still largely unknown, such as in, for example, in hickory (*Carya cathayensis* Sarg.), a unique woody oil tree species with high nutritional value and substantial health benefits (Huang et al., 2022).

Hickory, a perennial woody plant, is of major economic importance in temperate climate regions (Huang et al., 2019). However, it has a long juvenile phase and takes more than 10 years to bear fruit (Zheng et al., 2010). When the tree enters the reproductive stage, the male flower buds will begin to differentiate at the end of May of the previous year, and enter a dormant state after the primary stage of inflorescence axis; large bracts and small flowers are formed in mid-July (Zheng et al., 2012). The mid-May and mid-July of the same year is also when the fruit cattails volume and the fruiting branches of next year grow rapidly (Wang, 2006; Huang et al., 2013). Fierce competition for limited nutrients between vegetative and reproductive growth occur during this period (Shen et al., 2014). The fruits that drop peak in June of the same year reflect the consequences of both fierceness and competition (Wang, 2006). Though our team dived into the mechanisms of hickory buds’ differentiation, including the role of miRNAs (Wang et al., 2012; Wang et al., 2015; Sun et al., 2017), how lncRNAs may get involved remains unclear. Therefore, it is of vital importance to determine which lncRNAs play a role in hickory female bud differentiation.

In this study, we sought to identify lncRNAs from whole-transcriptomes of female flowers at different growth stages of hickory to understand lncRNAs function in floral development. A total of 6,862 putative lncRNAs were identified, and we predicted the target genes by location analysis. Our results revealed that lncRNAs might play an important role in phytohormone signaling during the hickory female flower differentiation period. In this work, we also predicted the function of lncRNAs, and the interaction network among lncRNAs, miRNAs, and genes. Overall, our investigation provides new preliminary insights for further research and evaluation of the molecular mechanisms of lncRNAs.

## Materials and Methods

### Plant Materials

The female flower buds at short pod-branches of hickory (ZAFU-1) from the nursery orchard of Zhejiang A&F University (lat. 30°15′N, long. 119°43′E), Zhejiang Province, China were collected for transcriptome sequencing. The sampling time was based on our previous scanning electron microscopy analysis and morphological observations of bourse shoot apices results (Huang et al., 2007). The female flower bud samples were collected at the undifferentiated stage (C1, March 11), differentiation stage (C2, March 18), and differentiation completed stage (C3, April 8) in 2016. All the collected flower buds were instantly frozen in liquid nitrogen and stored at −80°C for further usage.

### RNA Extraction, Library Construction, and Sequencing

Total RNAs were extracted from flower buds using the Total RNA Purification Kit (GeneMark, United Kingdom) following the manufacturer’s protocols. The RNA concentration and integrity were assessed using the NanoPhotometer^®^ spectrophotometer (IMPLEN, CA, United States) and the RNA Nano 6000 Assay Kit of the Bioanalyzer2100 system (Agilent Technologies, CA, United States), respectively.

A total of 3 μg per sample was used as starting material for the RNA sequencing (RNA-seq) libraries preparation. Ribosomal RNA was depleted using the Epicentre Ribo-Zero™ rRNA Removal Kit (Epicentre, United States), and rRNA free residue was cleaned up by ethanol precipitation. Subsequently, sequencing libraries were generated using the rRNA-depleted RNA by NEBNext^®^ UItra™ Directional RNA Library Prep Kit for Illumina ^®^(NEB, United States) following the manufacturer’s protocols.

The clustering of the index-coded samples was performed on a cBot Cluster Generation System using TruSeq PE Cluster Kit v3-cBot-HS (Illumia, United States) according to the manufacturer’s instructions. The clustered libraries were sequenced on an Illumina Hiseq 2500 platform, and 125 bp paired-end reads were generated.

### Identification of lncRNAs

Raw data (raw reads) in Fastq format, wherein reads containing an adapter, read ploy-N, and low-quality reads, were removed to obtain clean data (clean reads). The Q20 and Q30 percentages and GC content of the clean data were also calculated. An index of the hickory genome (obtained from the Juglandaceae genomic database, http://www.juglandaceae.net/) (Guo et al., 2020) was built using Bowtie (version 2.0.6) with default parameters (Langmead et al., 2009), and paired-end clean reads were aligned against the reference genome using TopHat (version 2.0.9) with parameters set as “--library-type” and “fr-firststrand” (Trapnell et al., 2009). After the alignment, the mapped reads of each sample was assembled in a reference-based approach using Scripture (beta2) (Guttman et al., 2010) and Cufflinks (version 2.1.1) (Trapnell et al., 2010). The default parameters were used for Scripture, while the parameters for Cufflinks were set as “min-frags-per-transfrag = 0”and “--library-type.” Cuffmerge (version 2.1.1,a package of Cufflink) was used to merge the assembled transcripts with default parameters (Trapnell et al., 2012). During the process, transcripts with uncertain strain direction were removed. Next, only transcripts with length ≥200 bp and exon numbers ≥2 were retained, and transcripts with low expression and confidence were filtered. The obtained transcripts were then compared with known lncRNAs using Cuffcompare (version 2.1.1, a package of Cufflink) (Trapnell et al., 2012) to get transcripts similar to the known lncRNAs. These transcripts were merged into the final lncRNA set for downstream analysis without further screening these transcripts. Then, with the help of Cuffquant (version 2.1.1, a package of Cufflink) (Trapnell et al., 2012), transcripts with the fragments per kilobase per million fragments mapped (FPKM) ≥ 0.5 were kept. After that, four calculator approaches: coding-non-coding-index (CNCI, version 2), coding potential calculator (CPC, version 0.9 -r2) (Kang et al., 2017), programmed frequency amplitude modulation (Pfam, version 1.3) (Bateman et al., 2002; Punta et al., 2012), and phylogenetic codon substitution frequency (PhyloSCF) (Lin et al., 2011), were used to screen for the transcripts that have protein-coding ability. Transcripts were predicted to have coding potential by any/all of the above four tools were filtered those without coding potential were putative lncRNA candidate data set.

### Expression Quantification and Differential Expression Analysis of Genes and lncRNAs

Cuffdiff (version 2.1.1, a package of Cufflink) (Trapnell et al., 2012) was used to calculate the FPKM of both genes and lncRNAs in each sample. The differential expression of genes and lncRNAs was determined using a model based on the negative binomial distribution of the datasets using Cuffdiff (Trapnell et al., 2010) with default parameters. Transcripts with a *p*-values < 0.05 were designated as differentially expressed.

### Real-Time Quantitative PCR

A total of 20 predicted lncRNAs, which were expressed in C1vsC2, C1vsC3, and C2vsC3, were selected for real-time quantitative RT-PCR (qRT-PCR). The primers were designed through the Primer-BLAST online tool (http://www.ncbi.nlm.nih.gov/tools/primer-blast/, accessed on 25 April 2018) and were listed in Supplementary Table S1. The cDNA was synthesized from 1 μg of total RNA in a 20 μL reaction mixture using the RevertAid First Strand cDNA Synthesis Kit (Thermo fisher, China) according to the manufacturer’s protocols. The qRT-PCR reaction mixture was prepared using Platinum®SYBR®Green qPCR SuperMix-UDG with ROX (Invitrogen, China) following the manufacturer’s protocols. The qRT-PCR was carried out on the CFX96 Real-Time System (BIO-RAD, CA, United States) with programs at 52°C for 2 min and 95°C for 2 min, followed by 40 cycles of 95°C for 15 s, 60°C for 30 s, and 72°C for 30 s. The expression level was normalized to the 18S rRNA gene of hickory, and the relative expression was calculated using the comparative Ct method (Schmittgen and Livak, 2008). All reactions were performed using one biological sample with three technical replicates.

### Target Gene Prediction and Enrichment Analysis

The potential target genes of differentially expressed (DE) lncRNAs were predicted based on their regulatory roles. Potential cis-acting target genes were essentially searched for by genome browsers and genome annotation close to lncRNAs within 100 kb upstream or downstream. Gene Ontology (GO) enrichment was performed using the GOseq (release2.12) R package, which corrected for gene length bias (Young et al., 2010) to gain insight into the functions of differentially expressed genes (DEGs) or lncRNA cis-acting target genes. GO terms with corrected P-adjust < 0.05 were considered enriched. KOBAS (version 2.0, http://www.genome.jp/kegg/) was used to test the statistical enrichment of DEGs or lncRNA target genes in Kyoto Encyclopedia of Genes and Genomes (KEGG) pathways (Kanehisa et al., 2008). The pathways with corrected P-adjust < 0.05 were determined as significantly enriched.

### Interaction Analyses of lncRNAs and/or Genes With miRNAs

The psRobot (version 1.2) (Wu et al., 2012) was used to predict whether an lncRNA could be an miRNA target. All the predicted miRNA sequences had their target gene identified using psRNA Target (release 2017) (Dai et al., 2018)with default parameters. The predicted genes that were targeted by miRNAs were annotated by Hmmscan (release 2.12) (Finn et al., 2011). The lncRNA-miRNA-gene interaction networks were constructed by combining the targeting relationships between miRNAs and lncRNAs. The possible regulatory relationships of lncRNA-miRNA-genes are shown in the network diagram and visualized using Cytoscape (version 3.7.0, http://www. cytoscape. org).

## Results

### Identification and Characterization of lncRNAs

We performed whole-transcriptome strand-specific RNA-seq from the female flower buds of the floral undifferentiated, differentiation, and differentiation complete stages, to identify long non-coding transcripts during female floral development of hickory. We obtained a total of 458,003,312 paired-end clean reads after filtering the adaptor sequences and low-quality reads from raw reads. Of which, approximately 81, 86, and 85% of these clean reads were mapped on hickory assembly, respectively (Table 1).

**TABLE 1:** Statistical data of the RNA-seq reads for three development stages.

Sample	Raw Reads	Clean Reads	Clean Bases (G)	Q20(%)	Q30(%)	GC Content(%)	Total Mapped
C1	164351770	157779036	23.67	94.71	88.13	47.75	127070560 (80.54%)
C2	154216020	147904658	22.19	94.66	87.92	48.59	127864069 (86.45%)
C3	158763504	152319618	22.85	94.67	88.03	47.06	129202584 (84.82%)

**FIGURE 1:**
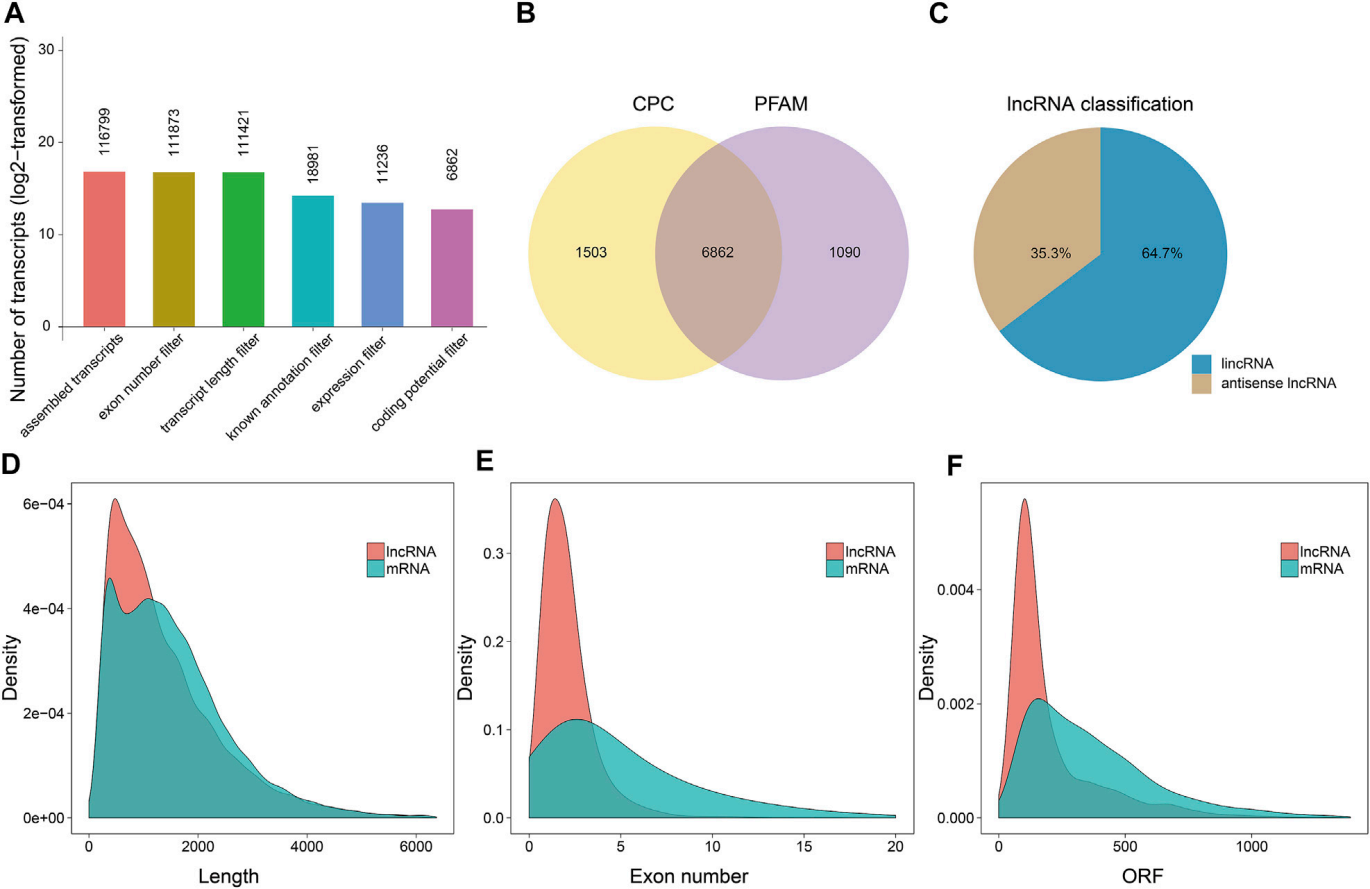
The characterization and identification of candidate lncRNAs. **(A)** Identification of lncRNAs from RNA sequencing data based on the structural characteristics of lncRNAs and the functional characteristics of non-coding proteins with five strict screening conditions. Each number represented the number of transcripts obtained after filtering. **(B)** The Venn diagram of potential candidate lncRNAs by competing with the Coding Potential Calculator (CPC) and the Programmed Frequency Amplitude Modulation (Pfam) database. **(C)** Classification of the predicted antisense and intergenic lncRNAs. **(D–F)** The length, exon number, and open reading frame (ORF) distribution of mRNAs and lncRNAs, respectively.

After merging and deleting the assembled transcripts by Cuffmerge, we obtained 116,799 transcripts (Figure 1A). We filtered the obtained transcripts according to their lengths, exon numbers, and FPKM expression level using Cuffcompare and Cuffquant. As a result, 11,236 transcripts passed the filtering stage. Next, we examined the coding potential of these transcripts using CPC (Kang et al., 2017) and Pfam database (Punta et al., 2012), and predicted a total of 6,862 candidate lncRNAs (Figure 1B). Of these 4,340 (64.7%) candidates were intergenic lncRNAs, and 2,522 (35.3%) were antisense lncRNAs (Figure 1C). We also noticed that the overall length of the lncRNAs and their open reading frames (ORFs) were shorter than those of the mRNAs (Figures 1D,F), and the exon number of the lncRNAs was less than that of the mRNAs (Figure 1E).

### Expression Profiles of Genes and lncRNAs During the Three Developmental Stages of Female Flowers

**FIGURE 2:**
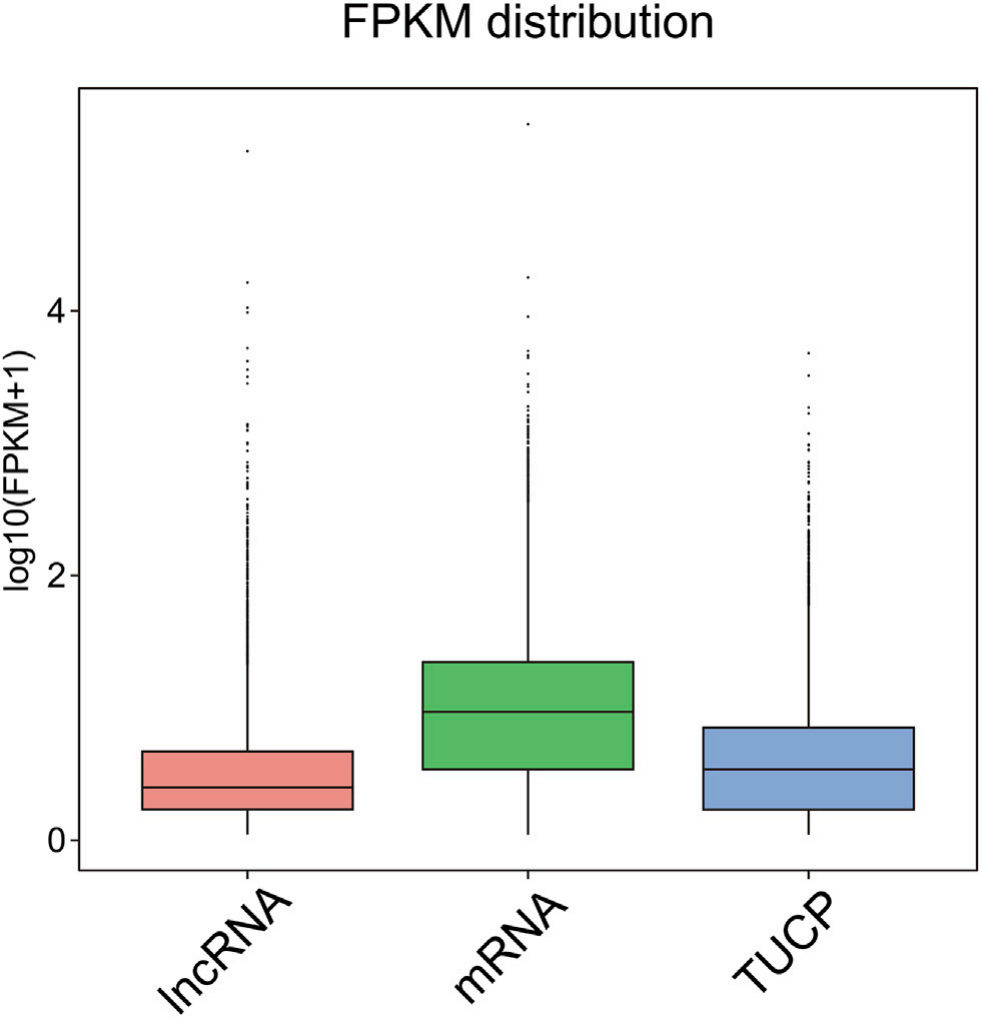
Box plots showing the expression levels of different types of transcripts. The horizontal coordinates indicate the different types of transcripts and the vertical coordinates are log10 (FPKM+1); there are box plots of each region for five statistics (maximum, upper quartile, median, lower quartile, and minimum).

After predicting putative lncRNAs, we used FPKM (Supplementary Tables S2, S3) to explore the expression profiles of the lncRNAs and associated protein-coding genes (mRNAs in this case). We noticed that the expression levels of lncRNA and transcripts of uncertain coding potential (TUCP) were lower than those of mRNAs (Figure 2). Then, we performed differential expression analyses of genes and lncRNAs at different developmental stages for female hickory flowers, and observed that the number of DE lncRNAs was less than that of DEGs.

**FIGURE 3:**
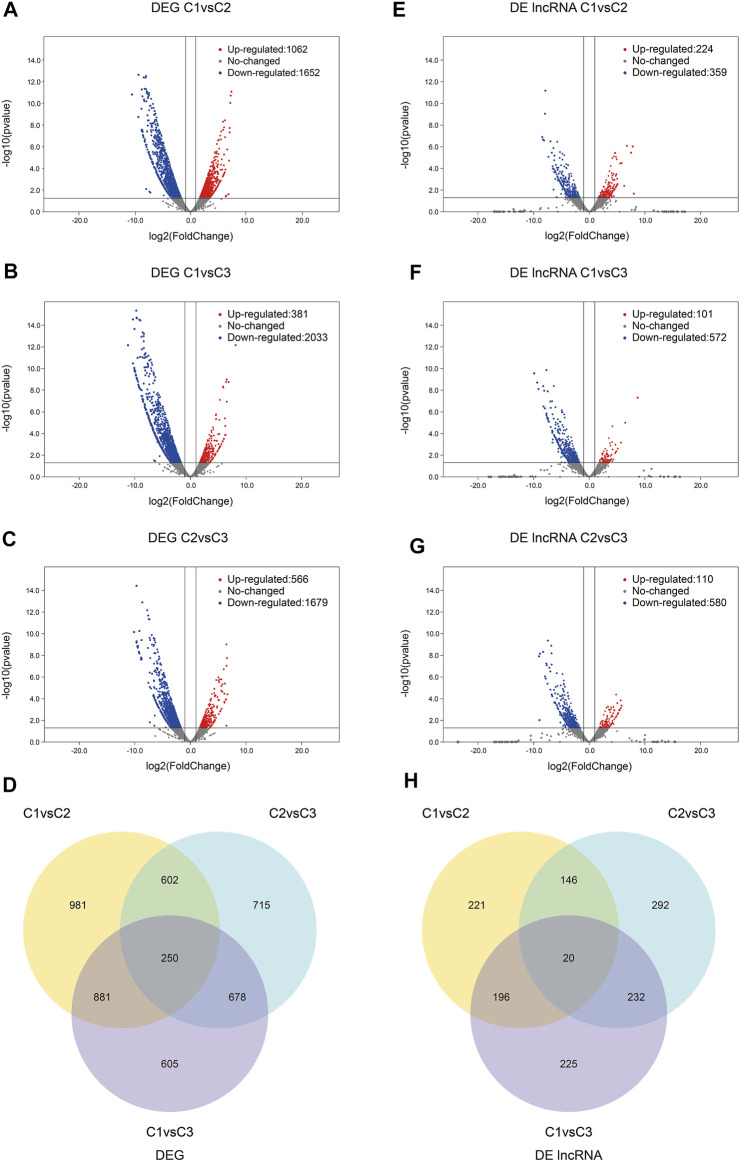
The differential expression of genes and lncRNAs. **(A–C)** Volcano plots of DEGs comparing C1vsC2, C1vsC3, and C2vsC3, respectively. Up-regulated genes were indicated by red dots, down-regulated genes were indicated by blue dots, and grey dots indicated constitutively expressed genes. **(D)** Venn Diagrams of all compared DEGs. **(E–G)** Volcano plots of the DE lncRNAs comparing C1vsC2, C1vsC3, and C2vsC3, respectively. Red and blue dots represented up- and down-regulated DE lncRNAs, respectively. The non-DE lncRNAs were represented by grey dots. **(H)** The Venn diagrams of the DE lncRNAs of all comparisons. C1, undifferentiated stage; C2, differentiation stage; C3, differentiation complete stage; DE, differentially expressed; DEG, differentially expressed gene.

**FIGURE 4:**
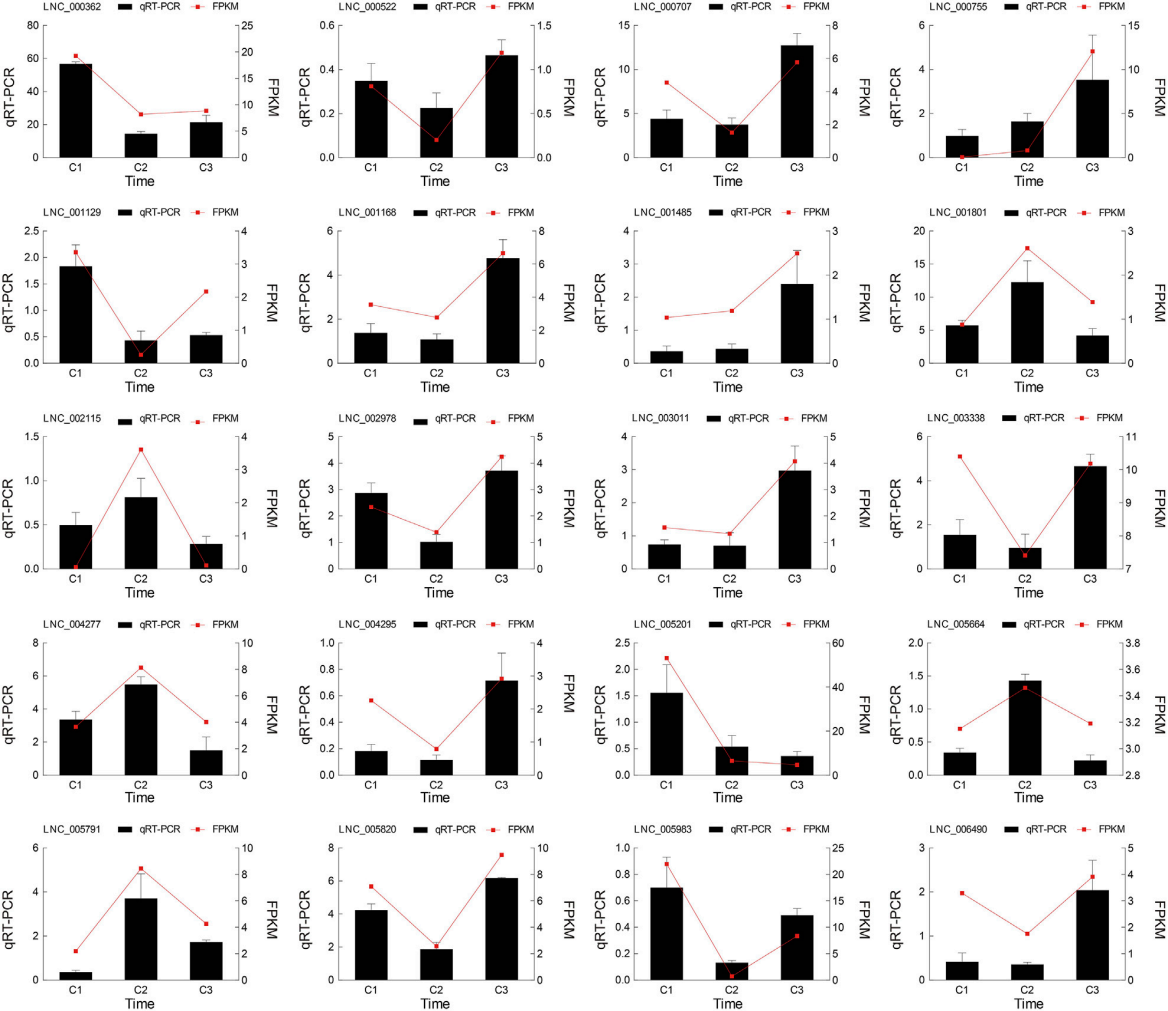
The comparison of the 20 lncRNAs’ real-time quantitative RT-PCR (qRT-PCR) and RNA sequencing (RNA-seq). Both datasets of hickory female floral development periods were in good agreement. The columns represented the results of the qRT-PCR, and the red lines represented the results of the RNA-seq.

Interestingly, the number of down-regulated genes and lncRNAs was greater than that of up-regulated genes and lncRNAs. The differential expressions of genes and lncRNAs were shown in volcano plots (Figure 3; Supplementary Tables S4, S5). We observed 250 prevalently differentially expressed genes in the C1vsC3, C2vsC3, and C1vsC2 differential expression analysis (Figure 3D). However, 20 lncRNAs were commonly differentially expressed in the three comparisons (Figure 3H). The expression patterns of DEGs and lncRNAs were presented in the heatmap in the Supplementary Figure S1. At the same time, we also compared the expression of the above 20 lncRNAs’ using qRT-PCR results with our RNA-seq dataset. The two datasets were in good agreement (Figure 4).

### Gene Ontology Enrichment Analysis of DEGs and DE lncRNA Cis-acting Target Genes

**FIGURE 5:**
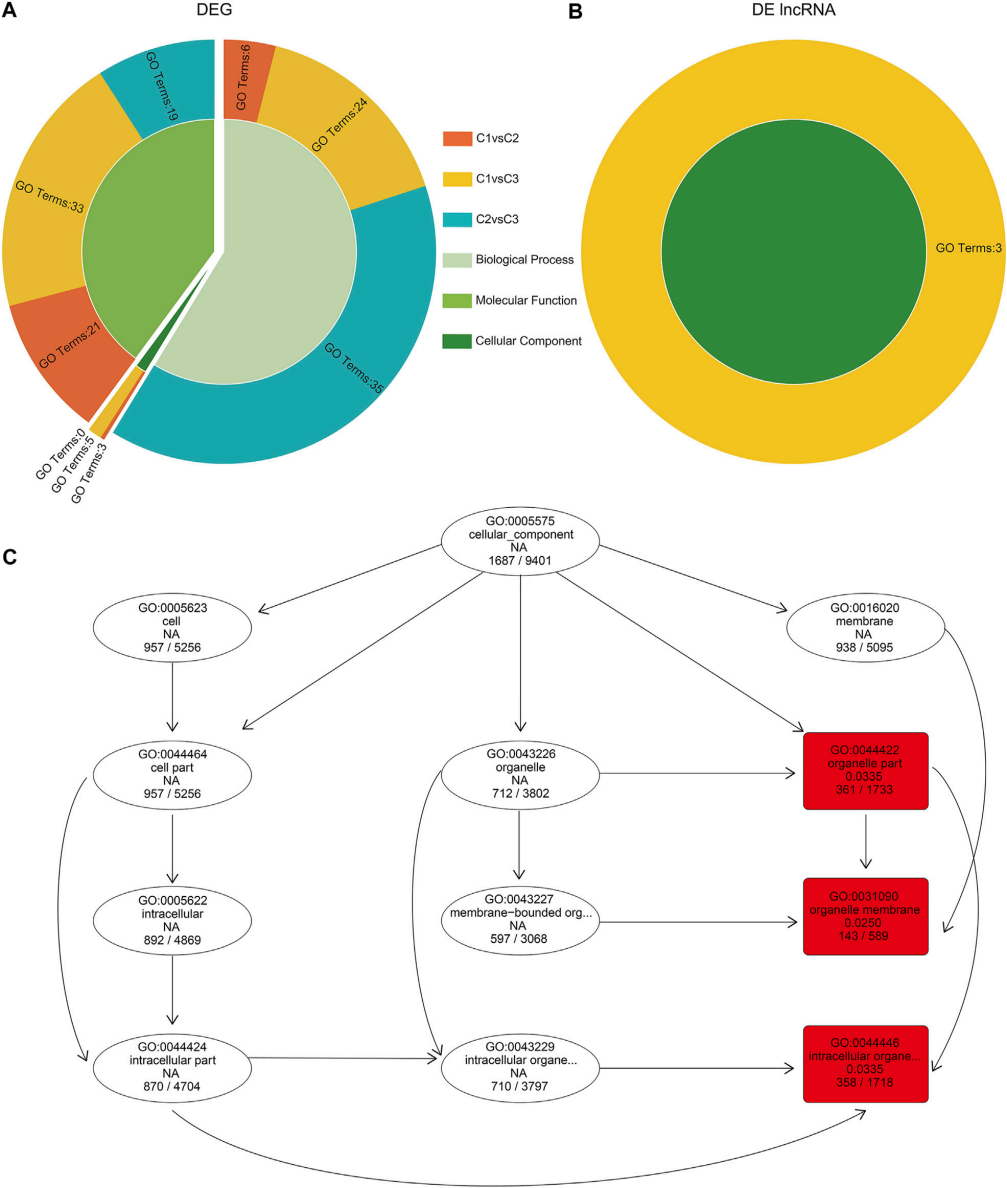
Gene Ontology (GO) enrichment analysis of DEGs and cis-acting target genes of DE lncRNA in hickory female floral development. **(A,B)** GO enrichment of DEGs and DE lncRNA cis-acting target genes in comparison to C1, C2, and C3 hickory female floral development. **(C)** The enriched GO terms (red boxes) for cis-acting target genes of lncRNAs in the cellular component of C1vsC3.

We performed GO enrichment analysis on the DEGs of undifferentiated, differentiated, and differentiated complete female buds. Overall, we noticed a similar number of genes enriched with the “biological process” and “molecular function” in all three comparisons. In contrast, the number of genes enriched with the “cellular component” was the least (Supplementary Table S6). For instance, in C2vsC3, no terms were enriched for the “cellular component.” However, in C1vsC2, the “cellular component,” including the “external encapsulating structure” (GO:0030312, 32 genes), “cell wall” (GO:0005618, 25 genes), and “apoplast” (GO:0048046, 12 genes), were enriched. Moreover, in C1vsC3, the “cellular component” enrichment term had more “cell periphery” (GO:0071944, 66 genes) and a greater “extracellular region” (GO:0005576, 96 genes) than in C1vsC2 (Supplementary Table S6, Figure 5A).

As lncRNAs regulate their target genes by cis-acting, we characterized the potential targets within the 100 kb upstream and downstream of DE lncRNAs, and obtained 24,507 genes for 6,018 lncRNAs (Supplementary Table S7). Interestingly, GO enrichment analysis of DE lncRNA target genes showed enrichment only in the cellular component in C1vsC3 (Supplementary Table S8; Figure 5B). Among them were “organelle part” (GO:0044422, 361 genes), “intracellular organelle part” (GO:0044446, 358 genes), and “organelle membrane” (GO:0031090, 143 genes, Figure 5C).

### Kyoto Encyclopedia of Genes and Genomes Enrichment Analysis of DEGs and DE lncRNA Cis-acting Target Genes

**FIGURE 6:**
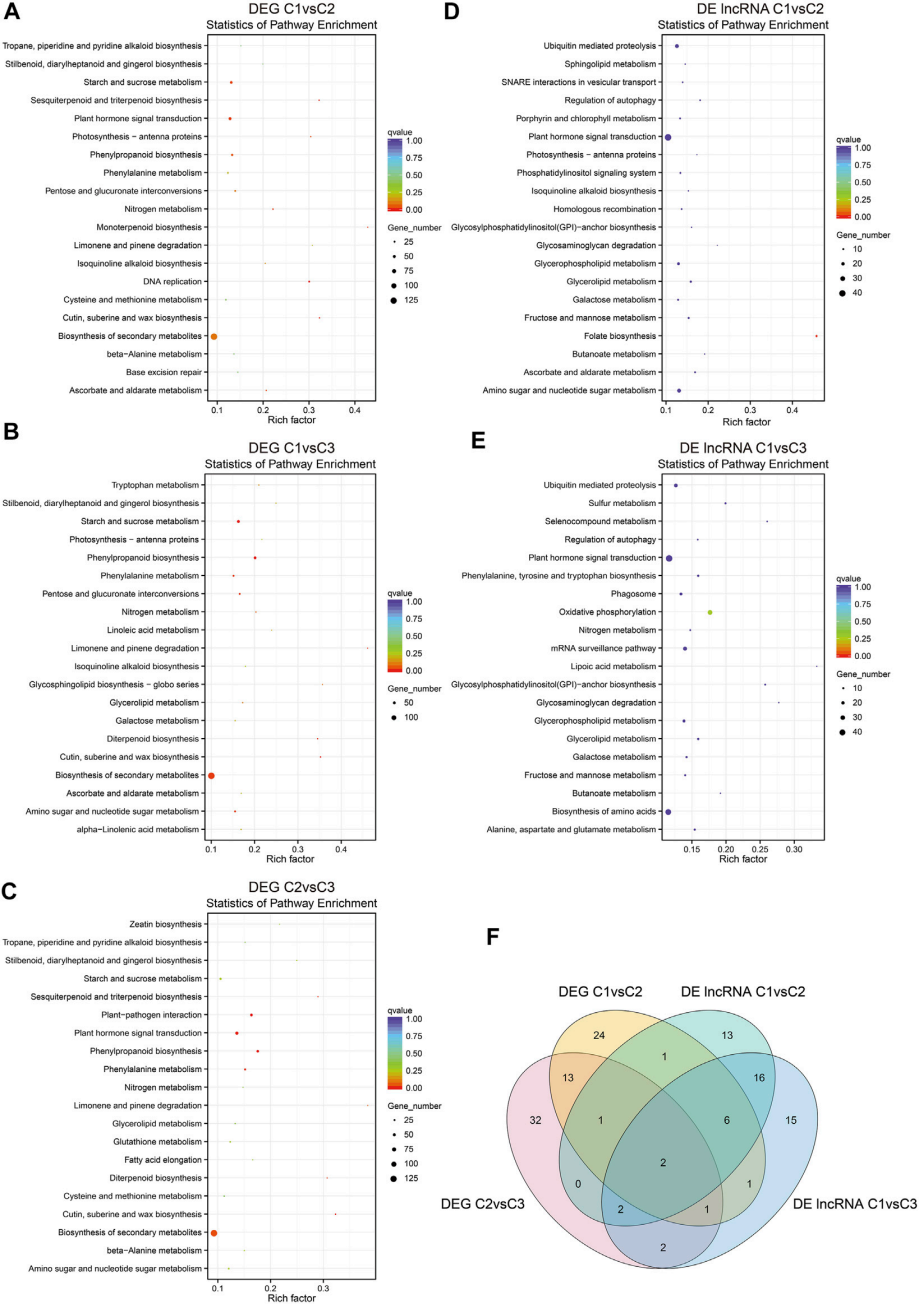
Kyoto Encyclopedia of Genes and Genomes (KEGG) enrichment pathway analysis of DEGs and DE lncRNA cis-acting target genes in hickory female floral development. **(A–C)** KEGG enrichment of DEGs in C1vsC2, C1vsC3, and C2vsC3. Only the top 20 enriched pathways were shown in the graphs. **(D,E)** The top 20 enriched pathways of the DE lncRNA cis-acting target genes in C1vsC2 and C1vsC3. **(F)** Venn diagram showing the number of unique and shared genes for DEGs and the cis-acting target genes of DE lncRNAs in the phytohormone signal transduction pathway in hickory female floral development. The *x*-axis and the *y*-axis represent the enrichment factors and the pathways, respectively. The different sizes of the dots indicate the number of genes in a pathway.

Our KEGG pathway enrichment analysis on the DEGs among C1vsC2, C1vsC3, and C2vsC3 indicated that they were enriched in cutin, suberine, and wax biosynthesis, and have a peak in biosynthesis in the secondary metabolites (Figures 6A–C). Plant hormone signal transduction was second to biosynthesis of the secondary metabolites, and was enriched in C1vsC2 and C2vsC3. On the other hand, the cis-acting target genes of DE lncRNAs in C1vsC2 and C1vsC3 were enriched, and folate biosynthesis was significantly enriched in C1vsC2. However, the plant hormone signal transduction pathway had the largest number of cis-acting target genes of DE lncRNAs (Figures 6D,E).

**FIGURE 7:**
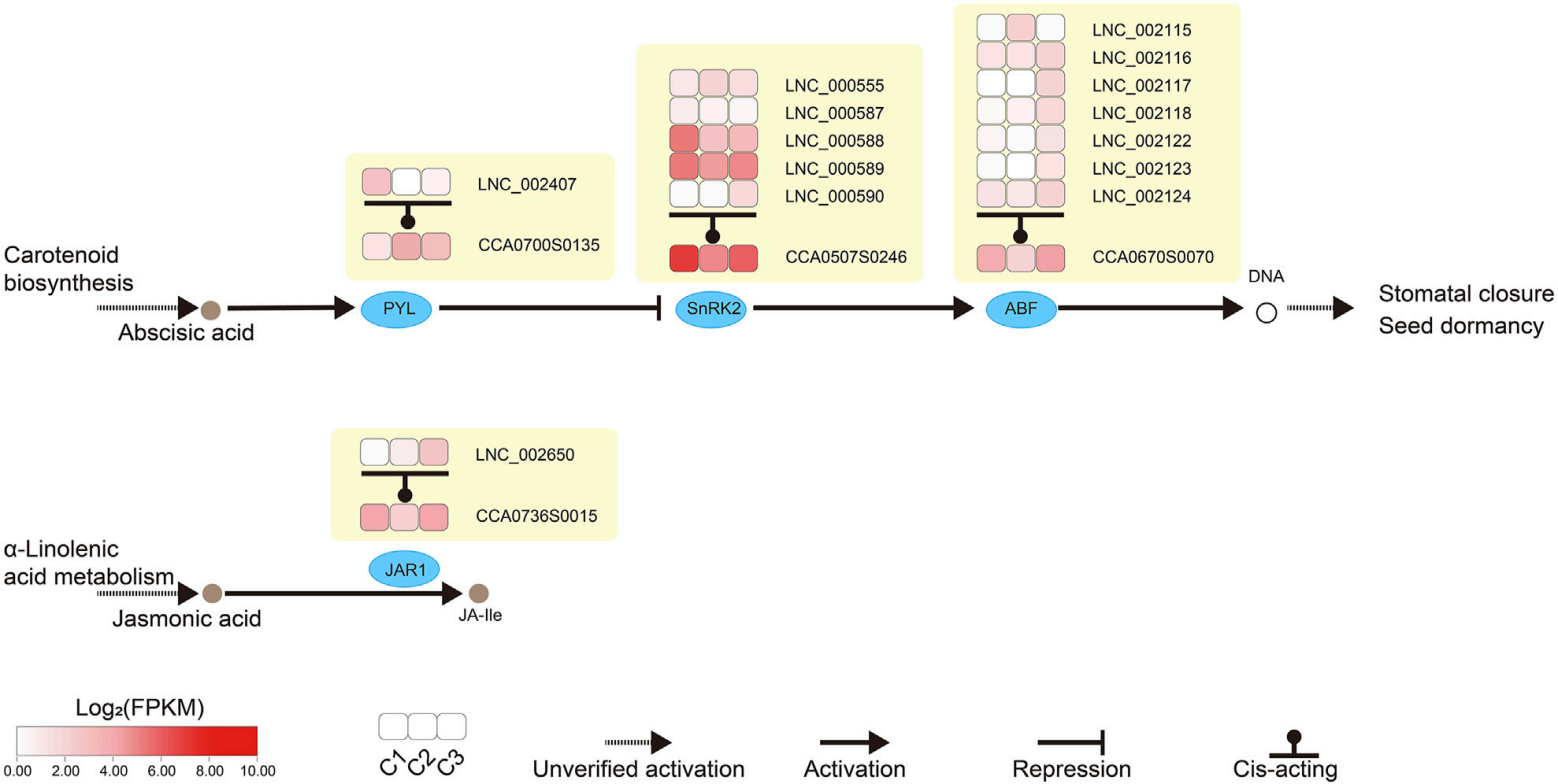
Analysis of lncRNAs involved in the abscisic acid and jasmonic acid signaling transduction pathways of hickory female flower buds. Heat maps in the boxes showed the expression level of lncRNAs (lncRNA identifier) and the target genes (CCA identifier) of these lncRNAs. In the Carotenoid biosynthesis pathway, the secretion of Abscisic acid promoted the expression of *PYL*, which in turn inhibited the expression of *SnRK2*, which was positively correlated with the expression of *ABF*. All three genes were regulated by lncRNA cis-acting, thereby affecting Stomatal closure and seed dormancy. In the *α*-Linolenic acid metabolism pathway, the production of JA-ile from jasmonic acid was affected by *JAR1*, which was also regulated by lncRNA in cis-acting. CCA, *Carya cathayensis*; *ABF, ABRE-binding factor; JAR1, JASMONATE RESISTANT 1; PYL, Pyrabactin resistance-like; SnRK2, SNF1-related protein kinase 2.*

We looked closely at the DEGs and cis-acting target genes of the lncRNAs involved in plant hormone signaling. We noticed that two genes were shared in both datasets during floral development (Figure 6F). One was *ABRE-binding factor (ABF)* in abscisic acid (ABA) signaling, and the other was *JASMONATE RESISTANT1 (JAR1)* in jasmonic acid signaling (Figure 7).

### Analysis of the lncRNA-miRNA-Gene ceRNA Network

**FIGURE 8:**
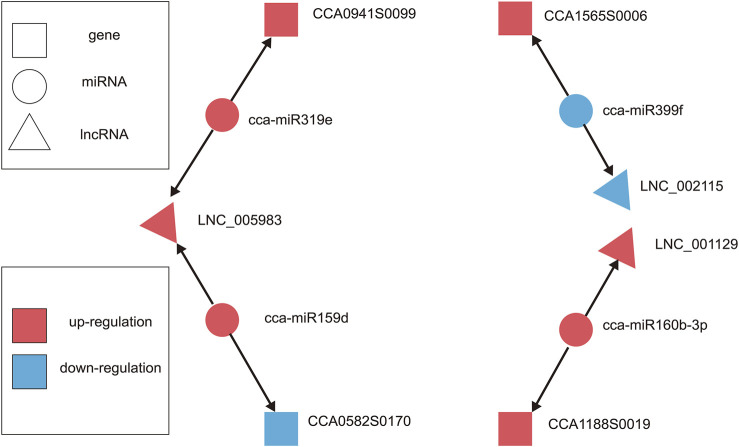
The hypothesized lncRNAs-miRNAs-gene expression interaction ceRNA network of hickory female floral development in C1vsC2. The triangle, circle, and square nodes denote lncRNAs, miRNAs, and genes, respectively. The miRNAs could affect the expression of lncRNAs and genes. The cca-miR159d and cca-miR319e affected the expression of LNC_005983, while the expression of CCA0941S0099 was promoted by cca-miR319e; CCA0582S170 expression was inhibited by cca-miR159d. There was also cca-miR399f, which positively regulated LNC_002115 but negatively regulated CCA1565S0006, while cca-miR160b-3p positively regulated lncRNA and gene. The red denotes up-regulation and the blue denotes down-regulation.

LncRNAs can function as endogenous miRNA sponges because they act as bait by pairing with miRNAs. This can competitively inhibit their interaction with mRNA, thereby regulating plant growth and development (Xu et al., 2016; He et al., 2018; Yang Z. et al., 2020). Interestingly, miRNAs targeting lncRNAs (miRNA-lncRNA) and miRNA-gene association analysis were predicted only in C1vsC2 for our results. We obtained five miRNA-lncRNA interactions that included flower-related miRNAs, such as miRNA159d, miRNA160a, miRNA160b-3p, miRNA319e, and miRNA399f (Supplementary Table S9). Our results also identified 36 miRNA-gene interactions, which included 20 known miRNA-gene interactions and 16 novel miRNA-gene interactions (Supplementary Table S10). Among them, miR159, miR160, miR167, miR169, miR319, and miR396 (miRNA families) were closely related to the growth and development of flowers (Bartel, 2009). We hypothesized that miRNA targets lncRNA and genes based on the ceRNA concept, and predicted the lncRNA-miRNA-gene expression interaction network of C1vsC2. A total of four differentially expressed miRNAs were targeted by three lncRNAs and four genes in the network (Table 2; Figure 8), where LNC_005983 could act as a sponge for cca-miR319e and cca-miR159d, thereby promoting CCA0941S0099 (unknown) while repressing *UDP-glucose iridoid glu-cosyltransferase-like* (*UDPGT*). LNC_001129 and *cellulose synthase-like protein H1* (*CLS-H1*) likely shared the same miRNA binding site and were regulated by cca-miR160b-3p. Interestingly, when the expression of cca-miR399f was down-regulated, the expression of LNC_002115 was also down-regulated, whereas the expression of *PHOSPHATE2* (*PHO2*) was up-regulated.

**TABLE 2:** CeRNA network and its target gene annotation in C1vsC2.

lncRNA ID	miRNA ID	Gene ID	Annotation
LNC_002115	cca-miR399f	CCA1565S0006	*PHOSPHATE2 (PHO2)*
LNC_005983	cca-miR159d	CCA0582S0170	*UDP-glucose iridoid glucosyltransferase-like (UDPGT)*
LNC_005983	cca-miR319e	CCA0941S0099	*unknown*
LNC_001129	cca-miR160b-3p	CCA1188S0019	*cellulose synthase-like protein H1 (CLS-H1)*

## Discussions

### Characteristics of lncRNAs Involved in Hickory Female Floral Development

In this study, we obtained 6,862 novel lncRNAs *via* whole-transcriptome analysis in three developmental stages of hickory female flowers (Supplementary Table S3). While analyzing the characteristics of hickory lncRNAs, we noticed that the exon length, the exon numbers, and ORF length of hickory were similar to those reported in *Cicer arietinum, Cucumis melo*, and *Prunus mume* (Khemka et al., 2016; Tian et al., 2019; Wu et al., 2019). The average length of lncRNAs was 1,459 bp, which was less than the average length of mRNAs (Figure 1D). In *Populus*, the average length of lncRNAs was half that of protein-coding transcripts and the expression level of lncRNA was lower than that of genes (Tian et al., 2016). The same results were observed in *P. mume* (Wu et al., 2019). However, lncRNAs expression is highly tissue-specific and cell type-specific in most plants (Li et al., 2014)*.* This could be a specific characteristic to perennial woody plants.

### Functional Enrichment of DEGs and DE lncRNA Cis-regulatory Target Genes

We found that C1vsC2 had the most DEGs and the least DE lncRNAs. LncRNAs can regulate target genes expression through multiple mechanisms; for example, they can act as miRNA decoys to activate gene expression by sequestering miRNA (Guttman and Rinn, 2012). In addition, lncRNAs can regulate target genes by acting in cis or trans (Wu et al., 2019). This work predicted that 6,018 lncRNAs may regulate 24,507 target genes by cis-acting, suggesting that one lncRNAs can regulate multiple genes (Supplementary Table S7).

**FIGURE 9:**
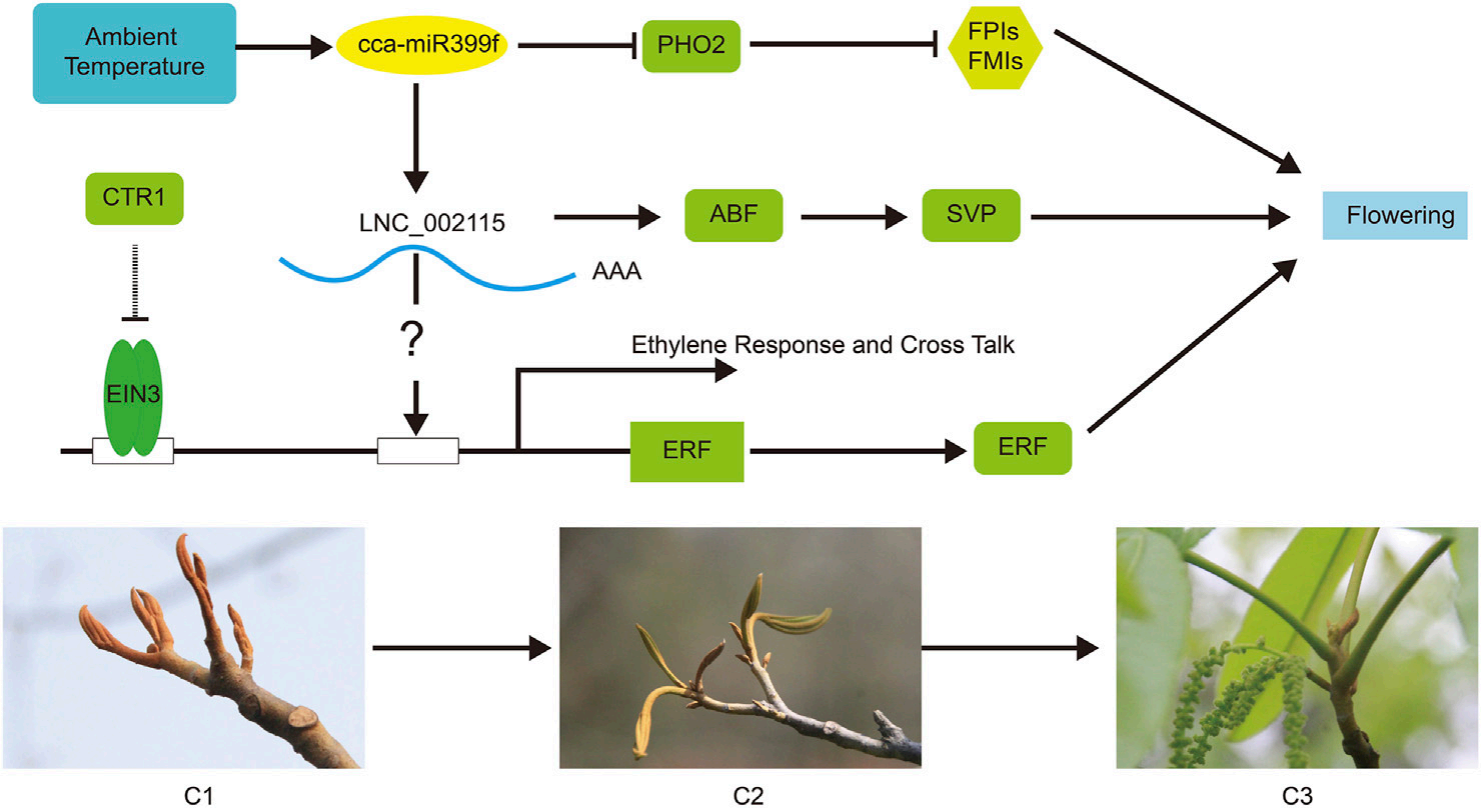
The competing endogenous RNA (ceRNA) regulation hypothetical model of hickory female floral bud development during the change of ambient temperature. The arrows indicate positive regulation, and blunt-ended bars indicate inhibition. A line does not necessarily represent unique or direct regulation. Change in ambient temperature may influence flowering by affect cca-miR399f to suppress the expression of *PHO*, thereby inhibiting the expression of floral meristem identity (FMI) and floral pathway integrator (FPI) genes. Meanwhile, cca-miR399f stimulated the expression of *ABF* and *SVP* by promoting the expression of LNC_002115, thereby affecting flowering. LNC_002115 might also interact with the *ERF* promoter to activate *ERF* expression and ultimately affect flowering. *ABF* (CCA0670S0070), *ABRE-binding factor*; *CTR1* (CCA0854S0091)*, Copper transporter 1; EIN3* (CCA0613S0076)*, ETHYLENE-INSENSITIVE3; ERF* (CCA0510S0116)*, Ethylene-responsive element-binding factor*; *PHO2* (CCA1565S0006)*, PHOSPHATE2; SVP* (CCA1068S0034)*, SHORT VEGETATIVE PHASE.*

Plant hormones are essential for most aspects of plant growth, differentiation, and development (Wu et al., 2019). In the KEGG pathway analysis, phytohormone signaling pathways were enriched in abscisic acid and jasmonic acid signaling (Figure 7). In *P. mume*, the lncRNA of TCONS_00032517 induced the expression of a cytokinin negative regulator gene, A-ARR, by inhibiting target miRNA in multiple pistils of Da Yu cultivar (Wu et al., 2019). A-ARR, together with TOCNS_00032517, was presumed to regulate floral development and affected pistil formation (Wu et al., 2019). A total of 16 DEGs and 52 DE lncRNAs were enriched for phytohormone signaling in our study. We also observed that multiple lncRNAs regulated some DEGs. Our findings also suggested that lncRNAs may regulate phytohormones and influence floral development (Figures 6F, 7). *ABF* in the ABA pathway played an important regulatory role in plant bud dormancy. In pear, endo-dormancy maintenance and the expression level of *Pyrus pyrifolia Dormancy-associated MADS-box 3* (*PpyDAM3)* were controlled by the ABA content in flower buds (Yang Q. et al., 2020). The expression of *P. pyrifolia ABREBINDING FACTOR3 (PpyABF3)* was positively correlated with *PpyDAM3* expression (Yang Q. et al., 2020). Our study suggests that LNC_002115 might affect ABA content by repressing *ABF* expression, ultimately breaking dormancy and promoting female flower opening after winter dormancy (Figure 9).

### The lncRNA-miRNA-Gene-Related Network in Floral Development

The miRNAs in plants are important regulatory members involved in various plant life processes (Jalali et al., 2013). Studies have found that some miRNAs played irreplaceable regulatory roles in flowering and floral organ development (Jin et al., 2013). LncRNAs can either act as precursors of miRNAs or compete with mRNAs for miRNA binding (Shin et al., 2006; Franco-Zorrilla et al., 2007). Our study identified five miRNA-lncRNA interactions and 36 miRNA-gene interactions in C1vsC2 (Supplementary Tables S9, S10). Given the above information, the essential functioned pattern relationships among lncRNAs, targeted miRNAs, and targeted genes (mRNAs) require further investigation. We constructed the lncRNA-miRNA-gene-related network of hickory flowers based on the ceRNA concept (Table 2; Figure 8). We speculated that LNC_001129 and CCA1188S0019 might have the same miRNA-binding site, and cca-miR160b-3p regulated both. Similarly, LNC_005983 and CCA0941S0099 likely have the same miRNA-binding site and were regulated by cca-miR319e. In *A. thaliana*, the *Induced by Phosphate Starvation 1 (IPS1)* acts as a sponge to sequester miR399 and reduce its availability to target and degrade *PHO2* mRNA (Franco-Zorrilla et al., 2007). These results suggested that the association of lncRNAs with miRNAs and genes indicated their vital functions in plants.

### LNC_002115 Regulates Hickory Female Floral Development in Facing Temperature Change

Moderate changes in ambient temperature can significantly affect flowering time (Kim et al., 2011). As the temperature rises, the female hickory flower bud (terminal bud that grows on the short pod branches) begins to differentiate after being released from hibernation in late March every year (Huang et al., 2013). A previous study has identified six ambient temperature-responsive miRNAs (miR156, miR163, miR169, miR172, miR398, and miR399) in *A. thaliana*. The miR399-*PHO2* modulated plant flowering time in response to changes in ambient temperature (Lee et al., 2010), with more abundant mature miR399 in plants, grew at 23°C than in plants grown at 16°C. The expression levels of *PHO2* at different temperatures were negatively correlated with the expression of miR399 (Kim et al., 2011). In *Citrus*, miR399 has a conserved role in Pi homeostasis and has novel functions in reproductive development, male sterility, and anther dehiscence (Wang et al., 2020).

Interestingly, in our study, LNC_002115 was predicted to be a “decoy” for hickory cca-miR399f/*PHO2*. The cca-miR399f affected flowering by repressing the expression of *PHO2*, whereas cca-miR399f promoted the expression of LNC_002115 (Figure 8). Our study also revealed that LNC_002115 affected the development of hickory female flowers by affecting the transcriptional regulation of *ERF*, thereby influencing the expression of *ERF* under changes in ambient temperature. LNC_002115 may target the expression of *ABF* and *SVP*, ultimately breaking dormancy and promoting female floral development. To better understand the complex mechanism of female floral development in hickory, we constructed a hypothetical model based on key data at the transcriptional level based on DEGs, DE lncRNAs expression, ceRNA theory, and previous studies (Lee et al., 2010; Kim et al., 2011; Wang et al., 2020) (Figure 9). Nonetheless, further studies are needed to investigate the links between changes in ambient temperature, hormone content, and the expression level of lncRNAs, miRNAs, and genes.

## Conclusion

In this work, we examined the expression of lncRNA during the hickory flowering stage and identified significant differences among lncRNAs in female hickory flowers. Characteristic and differential analysis indicated that different lncRNAs were produced during the development of female flowers to regulate plant growth and development through cellular processes and phytohormone signaling. Based on the ceRNA hypothesis, LNC_002115 was predicted to be a “decoy” of hickory cca-miR399f/*PHO2* under the change of ambient temperature. LNC_002115 could also regulate *ABF* expression and affect the transcriptional regulation of *ERF*, ultimately affecting female floral development. Our study revealed the functional patterns of lncRNAs, including the regulation of related target genes, serving as precursors of miRNAs, and competing with genes for binding to miRNAs. In conclusion, this study shed light on our understanding of the lncRNAs in the flowering process of hickory female flowers, and provides preliminary evidence for further research on the molecular mechanisms of the hickory flowering mechanism.

## Data Availability

The datasets presented in this study can be found in online repositories. The names of the repository/repositories and accession number(s) can be found below: https://www.ncbi.nlm.nih.gov/, PRJNA820165.
