# Supplementary material for: Whole-Transcriptome Analysis Reveals Long Noncoding RNAs Involved in Female Floral Development of Hickory (Carya cathayensis Sarg.)
Source: Front Genet. 2022 May 11;13:910488. doi: 10.3389/fgene.2022.910488 (PMC9130753; doi:10.3389/fgene.2022.910488)
Supplement: Supplementary file 9 [file Image1.PDF]

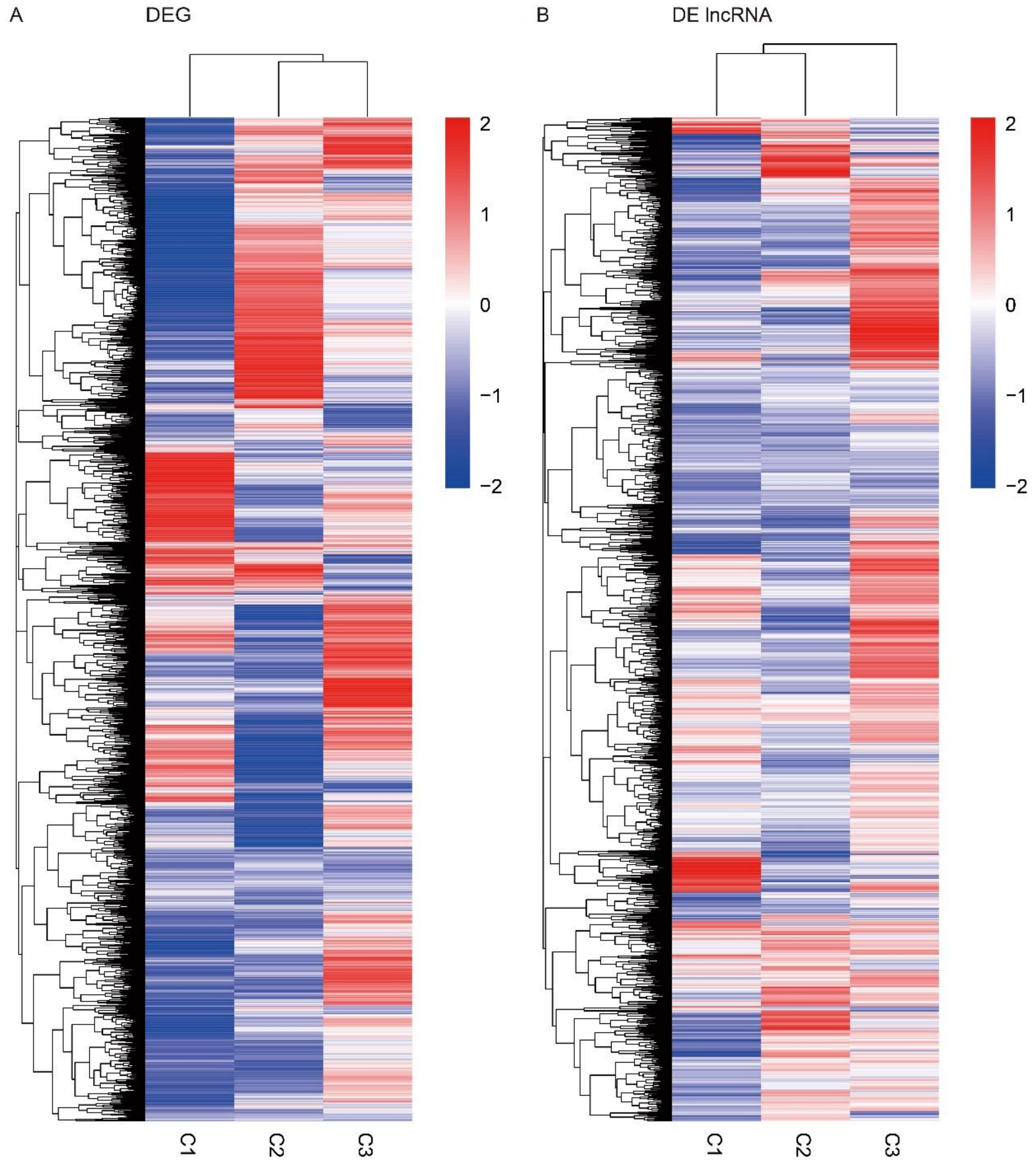

Supplementary Figure S1. Systematic cluster analysis of DEGs and DE lncRNAs in the three female floral development stages. A Clustering analysis of all the DEGs. Overall fragments per kilobase per million fragments mapped (FPKM) hierarchical clustering map with log10(FPKM+1) values for clustering, red indicates high expression genes and blue indicates low expression genes. B Clustering analysis of all the DE lncRNAs. Overall FPKM hierarchical clustering map with log10(FPKM+1) values for clustering, red indicates high expression lncRNAs and blue indicates low expression lncRNAs. C1, undifferentiated stage; C2, differentiation stage; C3, differentiation complete stage; DE, differentially expressed; DEG, differentially expressed genes
